# Influence of Dental Titanium Implants with Different Surface Treatments Using Femtosecond and Nanosecond Lasers on Biofilm Formation

**DOI:** 10.3390/jfb14060297

**Published:** 2023-05-26

**Authors:** Bo Yun Seo, KeunBaDa Son, Young-Tak Son, Ram Hari Dahal, Shukho Kim, Jungmin Kim, JunHo Hwang, Sung-Min Kwon, Jae-Mok Lee, Kyu-Bok Lee, Jin-Wook Kim

**Affiliations:** 1 Department of Oral & Maxillofacial Surgery, School of Dentistry, Kyungpook National University, Daegu 41940, Republic of Korea; seoboyun@naver.com; 2 Advanced Dental Device Development Institute (A3DI), Kyungpook National University, Daegu 41940, Republic of Korea; oceanson@knu.ac.kr (K.S.); dudxkr741@naver.com (Y.-T.S.); 3 Department of Dental Science, Graduate School, Kyungpook National University, Daegu 41940, Republic of Korea; 4 Department of Microbiology, School of Medicine, Kyungpook National University, Daegu 41944, Republic of Korea; ramhari.dahal@knu.ac.kr (R.H.D.); shukhokim@knu.ac.kr (S.K.); minkim@knu.ac.kr (J.K.); 5 Institute of Advanced Convergence Technology, Kyungpook National University, Daegu 41061, Republic of Korea; hjh@iact.or.kr (J.H.); sungmin@iact.or.kr (S.-M.K.); 6 Department of Periodontology, School of Dentistry, Kyungpook National University, Daegu 41940, Republic of Korea; leejm@knu.ac.kr; 7 Department of Prosthodontics, School of Dentistry, Kyungpook National University, Daegu 41940, Republic of Korea

**Keywords:** dental implants, surface treatment, surface roughness, biofilm formation, femtosecond laser, nanosecond laser

## Abstract

This study aimed to evaluate the impact of different surface treatments (machined; sandblasted, large grit, and acid-etched (SLA); hydrophilic; and hydrophobic) on dental titanium (Ti) implant surface morphology, roughness, and biofilm formation. Four groups of Ti disks were prepared using distinct surface treatments, including femtosecond and nanosecond lasers for hydrophilic and hydrophobic treatments. Surface morphology, wettability, and roughness were assessed. Biofilm formation was evaluated by counting the colonies of *Aggregatibacter actinomycetemcomitans* (Aa), *Porphyromonas gingivalis* (Pg), and *Prevotella intermedia* (Pi) at 48 and 72 h. Statistical analysis was conducted to compare the groups using the Kruskal–Wallis H test and the Wilcoxon signed-rank test (α = 0.05). The analysis revealed that the hydrophobic group had the highest surface contact angle and roughness (*p* < 0.05), whereas the machined group had significantly higher bacterial counts across all biofilms (*p* < 0.05). At 48 h, the lowest bacterial counts were observed in the SLA group for Aa and the SLA and hydrophobic groups for Pg and Pi. At 72 h, low bacterial counts were observed in the SLA, hydrophilic, and hydrophobic groups. The results indicate that various surface treatments affect implant surface properties, with the hydrophobic surface using femtosecond laser treatment exerting a particularly inhibitory effect on initial biofilm growth (Pg and Pi).

## 1. Introduction

The use of dental implants, which serve as artificial tooth roots to support dental prostheses, has become a widely accepted and effective treatment option to replace missing teeth [1,2,3]. Despite their high success rates, the long-term stability of dental implants can be jeopardized by bacterial colonization and biofilm formation [4]. These factors can lead to peri-implantitis, a destructive inflammatory process that may result in implant failure [5]. Consequently, understanding and optimizing the properties of implant surface treatments to minimize bacterial adhesion and biofilm formation is crucial to the enhancement of the clinical outcomes of dental implant treatments [6]. Furthermore, the transmucosal portion of the implant should provide a surface that inhibits biofilm formation while inducing soft tissue attachment [7].

Biofilms are complex microbial communities that adhere to surfaces and are surrounded by a self-produced extracellular polymeric matrix [8,9,10]. A biofilm formed along the mucous membrane of the peri-implant tissue is highly related to peri-implantitis [11]. Different biofilm types can be formed by various bacterial species, with each exerting distinct effects on peri-implantitis and other implant-related complications [12]. For example, *Aggregatibacter actinomycetemcomitans* (Aa) is a Gram-negative facultative anaerobe implicated in aggressive periodontitis, whereas *Porphyromonas gingivalis* (Pg) and *Prevotella intermedia* (Pi) are both Gram-negative anaerobes associated with chronic periodontitis and peri-implantitis [13]. Understanding how different surface treatments affect the formation of these biofilms is essential for the prevention of peri-implantitis and the improvement of dental implant longevity [14].

Laser technology, specifically femtosecond and nanosecond lasers, has emerged as a promising tool for surface treatment applications in the field of biomedicine including dental implants, corneal surgery, and tissue engineering [15]. The advantages of these lasers include high precision, minimal thermal damage, and the ability to create complex micro- and nanoscale surface features, allowing for precise and controlled energy delivery to the targeted surfaces [16]. Femtosecond lasers excel in generating intricate structures owing to their extremely short pulse durations, enhancing cellular responses and promoting better integration with biological tissues [17]. On the other hand, nanosecond lasers provide high levels of precision and control despite their longer pulse durations, making them suitable for various biomedical applications [18]. In addition, they tend to be more cost effective and offer a wider range of available wavelengths for diverse surface treatment applications [19]. These advanced laser systems represent highly valuable tools in surface treatment, allowing for precise and controlled modifications to materials within the field of biomedicine [20].

Surface treatments are essential for modifying dental implant surfaces to improve biocompatibility and osseointegration—the direct structural and functional connections between living bone and the implant surface [21,22]. Treatments can alter surface properties, such as roughness and hydrophilicity/hydrophobicity, which, in turn, influence bacterial adhesion and biofilm formation [23]. Traditional surface treatments, such as machined and sandblasted, large grit, and acid-etched (SLA), have achieved promising results in promoting cell attachment, proliferation, and differentiation, ultimately leading to improved osseointegration [24]. However, they also have drawbacks, such as increased bacterial adhesion and biofilm formation, potentially jeopardizing the long-term success of dental implants [25].

Innovative techniques such as femtosecond and nanosecond laser treatments have emerged as promising alternatives for implant surface modification [26]. This state-of-the-art approach allows for the creation of precise micro- and nanoscale surface patterns, which may yield unique surface properties and additional advantages compared with traditional surface treatments [26]. Potential benefits include enhanced osseointegration, reduced bacterial adhesion, and increased resistance to biofilm formation [27]. Despite its potential, research to explore the impact of femtosecond and nanosecond laser treatments on biofilm formation and bacterial colonization on dental implants, especially considering the distinct effects of different types of biofilms on peri-implantitis, is still lacking.

This study aimed to investigate how various surface treatment methods, including femtosecond and nanosecond laser treatments, influence surface morphology, roughness, and biofilm formation on dental titanium (Ti) implant disks. In addition, it aimed to compare the effects of machined, SLA, hydrophilic, and hydrophobic surface treatments on implant surface properties and their subsequent influence on biofilm formation involving Aa, Pg, and Pi. This study hypothesized that the machined, SLA, hydrophilic, and hydrophobic surface treatment methods of dental Ti implant disks do not affect surface morphology, roughness, and biofilm formation (Aa, Pg, and Pi, which are associated with peri-implantitis).

## 2. Materials and Methods

### 2.1. Surface Treatment of Titanium Disks

The Ti disk fabrication and SLA process were performed by a dental implant manufacturer (DENTIS; Daegu, Republic of Korea). The composition of the Ti disks (grade 4) was as follows: 0.08% carbon (C), 0.5% iron (Fe), 0.015% hydrogen (H), 0.05% nitrogen (N), 0.40% oxygen (O), and 98.9% Ti. Because of proprietary concerns, obtaining detailed information regarding the technology from the manufacturer is challenging. The grade 4 Ti disks (10 mm diameter and 1 mm thickness) were subjected to a machining process using a CNC milling machine to create a smooth surface with minimal roughness. Cutting parameters and tools were selected to minimize surface defects and achieve the desired surface finish. The Ti disks manufactured through this process were designated as the machined surface group.

The Ti disks were blasted with large grit alumina (250–500 µm) particles using a sandblasting machine at a pressure of 4 bar for 20 s. Subsequently, the disks were acid-etched in a mixture of HCl and H_2_SO_4_ (1:1 *v*/*v*) for 30 min at 60 °C, followed by rinsing with distilled water and drying at room temperature. The Ti disks manufactured through this process were designated as the SLA surface group.

**Figure 1:**
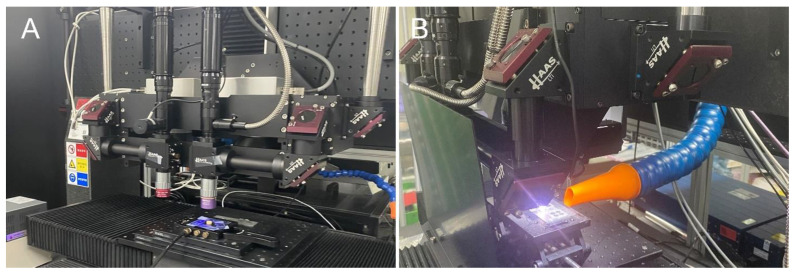
Laser systems for hydrophilic and hydrophobic surface treatments. (**A**): femtosecond laser system. (**B**): Nanosecond laser system.

In the present study, the modification of milled Ti disk surfaces to exhibit hydrophilic properties was investigated using an ultrashort pulse multiwavelength laser (Figure 1, Table 1). Specifically, a laser with a 343 nm wavelength was applied with a scanning speed of 10 mm/s and a repetition rate of 200 kHz to create a line pattern with a 50-µm pitch distance on both Ti and ceramic specimens (Figure 1, Table 1). The Ti disks fabricated through this process were categorized as the hydrophilic surface group.

In addition, to alter the surface of milled Ti disks to exhibit hydrophobic properties, a compact ultraviolet laser for process development was used (Figure 1, Table 1). A laser with a 355 nm wavelength was applied with a scanning speed of 100 mm/s and a repetition rate of 120 kHz to create a grid pattern with a 200-µm pitch distance on both Ti and ceramic specimens (Figure 1, Table 1). The specimens were then subjected to heat treatment in an oven for 2 h. The Ti disks fabricated through this process were designated as the hydrophobic surface group.

All specimens were post-processed in accordance with the protocols of the implant manufacturer (DENTIS; Daegu, Republic of Korea). The specimens underwent a standard washing procedure and packaging, after which sterilization was performed in compliance with the established protocol.

### 2.2. Wettability Evaluation

The wettability of the treated Ti disks was evaluated using contact angle measurements with a contact angle goniometer (Phoenix-MT; SEO, Suwon, Republic of Korea). A 2 µL droplet of deionized water was placed on each sample using a micro syringe, and the contact angle between the liquid and solid surfaces was measured within 10 s. The average contact angle was calculated from five measurements on each sample (N = 10 per surface treatment group). To account for the potential impact of surface roughness on wettability measurements, the surface roughness of each sample was considered when interpreting contact angle data. This approach helped to minimize possible distortions caused by surface topography.

### 2.3. Scanning Electron Microscopy and Confocal Scanning Microscopy

The Ti disks were cleaned in an ultrasonic bath with a mixture of acetone, ethanol, and distilled water (1:1:1 *v*/*v*/*v*) for 15 min each, followed by oven-drying at 60 °C for 2 h. The treated Ti disks were sputter-coated with a thin layer of gold–palladium (HPC-1SW; Vacuum Device Inc., Mito, Japan) and imaged using a scanning electron microscope (SEM; Hitachi SU8230; Hitachi, Tokyo, Japan) at an accelerating voltage of 5 kV and magnification of 100×. Confocal scanning microscopy images were acquired using a laser scanning confocal microscope (LEXT OLS4100; Olympus, Tokyo, Japan) at a magnification of 10×. The surface roughness of the treated Ti disks was quantitatively assessed by measuring the arithmetical mean roughness (Ra) and maximum roughness (Rz) parameters using the confocal scanning microscopy images. A 4 mm evaluation length was used for each measurement, and three measurements were performed on each sample. The data obtained were used to compare the effects of different surface treatments on implant surface roughness.

### 2.4. Bacterial Strains and Culture Conditions

Three bacterial strains, Aa KCOM 1299, Pg KCOM 2804, and Pi KCOM 3675, were obtained from the Korean Collection for Oral Microbiology and Department of Oral Biochemistry (Chosun University, Gwangju, Republic of Korea). All the strains were cultivated in modified brain heart infusion broth (32 g/L) supplemented with yeast extract (5 g/L; MBCell Kisanbio, Seoul, Republic of Korea), L-cysteine HCl (0.5 g/L; MBCell Kisanbio, Seoul, Republic of Korea), hemin solution (Sigma-Aldrich, St. Louis, MO, USA; final concentration 5 μg/mL), and vitamin K3 (menadione, Sigma-Aldrich, St. Louis, MO, USA; final concentration 1 μg/mL) at 37 °C for 24–48 h under anaerobic conditions with 5% CO_2_, 5% H_2_, and 90% N_2_ (Bactron Anaerobic Chamber, Seldon Manufacturing Inc., Cornelius, OR, USA). For the agar plates, 1.5% agar and 5% defibrinated sheep blood were supplemented.

### 2.5. Biofilm Development

For biofilm development, the Ti disks (machined, SLA, hydrophilic, and hydrophobic) were submerged in 24-well culture plates (Corning Inc., New York, NY, USA) containing 2 mL culture medium with each bacterial culture of Aa, Pg, and Pi (OD600 = 1.0). Subsequently, the disks were incubated at 37 °C for 48 and 72 h, respectively, under anaerobic conditions, with each treated surface of the disks in the upright position.

### 2.6. Bacterial Viability Assay

After 48 and 72 h, each Ti disk was gently washed with phosphate-buffered saline (PBS) three times. Then, the 1/3 part of each disk was washed with 70% ethyl alcohol to kill the bacteria present at the bottom surfaces of the disks (placing each disk in 12-well culture plates [Corning Inc., New York, NY, USA] containing 1 mL of 70% ethanol for 1 min, submerging the 1/3 part of the disks). Again, the 1/3 part of each disk was gently washed with PBS three times. Then, each disk was placed in a self-sealing sterilization pouch containing 5 mL PBS and then sonicated in ice-cooled water for 5 min in a sonicator (industrial analog ultrasonic cleaner SD-80W, Mujigae, Seoul, Republic of Korea) to detach the bacteria from the disks. After sonication, each disk was placed in 12-well culture plates (Corning Inc., New York, NY, USA) containing 1 mL PBS and sent for biofilm examination. Then, the PBS solutions were transferred into 15-mL conical tubes, serially diluted up to 10–3, plated on agar plates as previously described, and incubated anaerobically at 37 °C for 72 h. The number of colony-forming units (CFU) from each treatment was counted, and viable bacteria were determined (N = 5 per surface treatment group). All tests were conducted in duplicates.

### 2.7. Statistical Analysis

The number of samples utilized in this research was chosen based on a power analysis (G*Power version 3.1.9.2; Heinrich-Heine-Universität Düsseldorf, Düsseldorf, Germany) to ensure that the study was adequately powered to detect statistically significant differences between the treatment groups. The statistical analysis for this study was conducted using SPSS version 26 (IBM Corp., Armonk, NY, USA). Nonparametric tests were employed in the analysis due to the nature of the data. To ascertain the significant differences in roughness and surface contact angle among the four implant surface treatments, the Kruskal–Wallis H test was conducted (α = 0.05). In instances where significant differences were observed, pairwise comparisons were performed using the Bonferroni correction to account for multiple comparisons, and significant distinctions among the different implant surface treatments were denoted by distinct capital letters (α = 0.05). To identify the significant differences in viable bacterial counts on distinct implant surface treatments, the Kruskal–Wallis H test was employed (α = 0.05). Analogous to the roughness analysis, pairwise comparisons were conducted using the Bonferroni correction, and significant distinctions among the four implant surface treatments were denoted by distinct capital letters (α = 0.05). To evaluate the significant differences in viable bacterial counts between 48 and 72 h, the Wilcoxon signed-rank test was employed (α = 0.05).

## 3. Results

**Figure 2:**
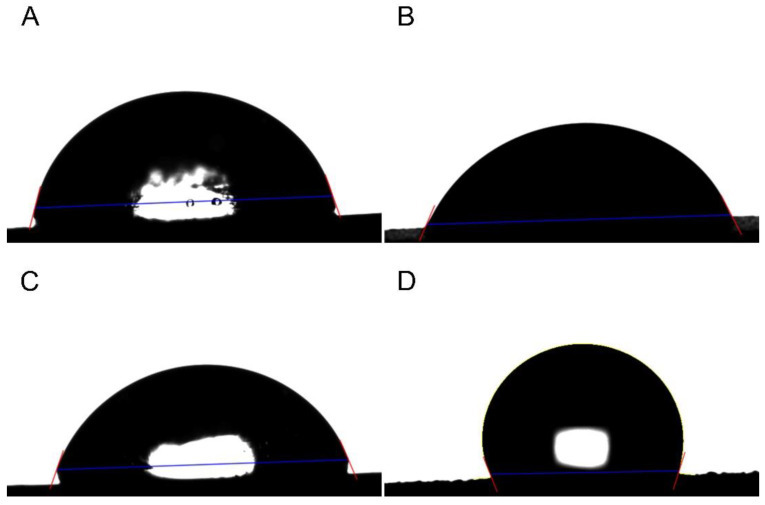
Representative surface contact angle image. (**A**): Machined group. (**B**): SLA group. (**C**): Hydrophilic group. (**D**): hydrophobic group.

The surface contact angle significantly varied among the different implant surface treatments (Table 2, Figure 2) (*p* < 0.001). The SLA and hydrophilic surfaces had similar surface contact angles (*p* > 0.05), and the hydrophobic surface had the highest surface contact angles (Table 2, Figure 2) (*p* < 0.05).

**Figure 3:**
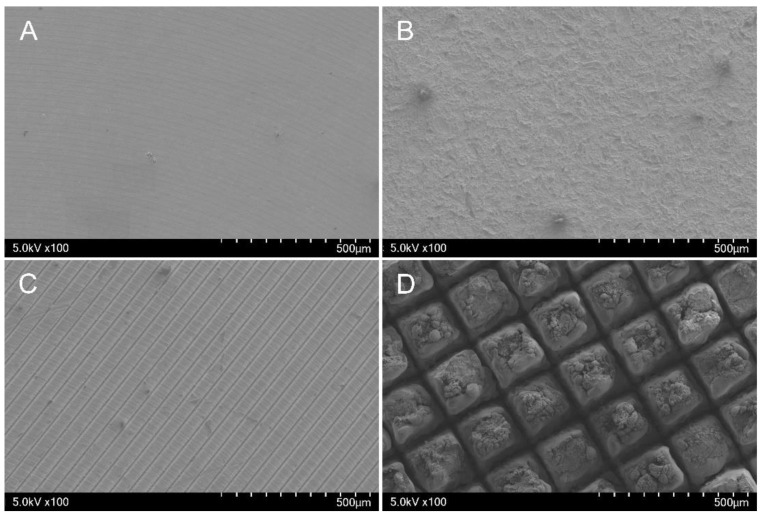
Representative surface morphology image using SEM. (**A**): Machined group. (**B**): SLA group. (**C**): Hydrophilic group. (**D**): Hydrophobic group.

In each group, the surface morphology of the samples was observed after disk surface treatment using SEM (Figure 3). The machined group exhibited a surface with machining traces on the disk (Figure 3A). The SLA group exhibited micropits formed by sandblasting to create roughness on machined Ti disks, followed by acid etching (Figure 3B). The hydrophilic group exhibited regular and repetitive parallel lines on the Ti disks, which were created by femtosecond laser treatment to achieve hydrophilicity (Figure 3C), whereas the hydrophobic group exhibited a repetitive lattice pattern on the Ti disks formed using femtosecond laser treatment for hydrophobicity (Figure 3D).

**Figure 4:**
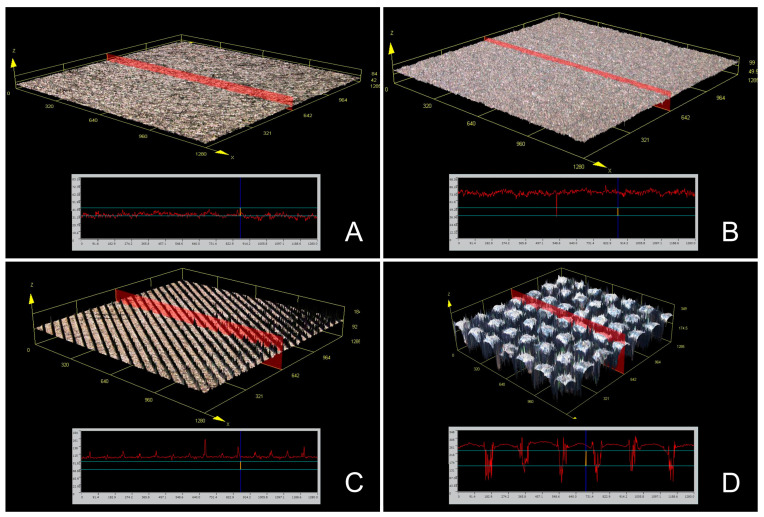
Representative surface morphology image using confocal laser scanning microscopy. (**A**): Machined group. (**B**): SLA group. (**C**): Hydrophilic group. (**D**): Hydrophobic group.

Confocal scanning microscopy was adopted to examine the surface roughness and sample surface morphology in each group (Figure 4). The same sample surface morphologies as observed in the SEM results were noted (Figure 4). The disk surface roughness (Ra, Rz) varied depending on the surface treatment group (Table 3) (*p* < 0.001), with the hydrophobic group exhibiting significantly higher surface roughness (Ra, Rz) than the other groups (Table 3) (*p* < 0.05). The machined, Ti SLA, and hydrophilic groups had similar surface roughness (Ra, Rz) (Table 3) (*p* > 0.05).

A significant difference was observed in the number of bacteria (CFU/mL) in the biofilm formed within 48 h, depending on the disk surface treatment group (Table 4) (*p* < 0.001). Differences were observed in the biofilms of Aa, Pg, and Pi, depending on the disk surface treatment group (Table 4) (*p* < 0.001). For Aa, the SLA group had the lowest bacterial count, whereas the machined group had the highest (Table 4) (*p* < 0.05). For Pg, both the SLA and hydrophobic groups had the lowest bacterial counts, whereas the machined group had the highest (Table 4) (*p* < 0.05). For Pi, both the SLA and hydrophobic groups had the lowest bacterial count, whereas the machined group had the highest (Table 4) (*p* < 0.05).

A significant difference was also observed in the number of bacteria (CFU/mL) in the biofilm formed within 72 h, depending on the disk surface treatment group (Table 5) (*p* < 0.001). Differences were observed in the biofilms of Aa, Pg, and Pi, depending on the disk surface treatment group (Table 5) (*p* < 0.001). Unlike the 48 h results, all biofilms had similar bacterial counts in the SLA, hydrophilic, and hydrophobic groups (*p* > 0.05), whereas the machined group had the highest bacterial count (Table 5) (*p* < 0.05).

Comparing the biofilm surviving bacterial counts between 48 and 72 h, a significant increase in bacterial counts was observed in most disk surface treatment groups at 72 h (Table 5) (*p* < 0.05). No significant increase in the bacterial counts was observed at 72 h in Aa for the hydrophilic group (*p* = 0.972), Pg for the SLA group (*p* = 0.092), and Pi for the hydrophilic group (*p* = 0.734) (Table 5).

**Figure 5:**
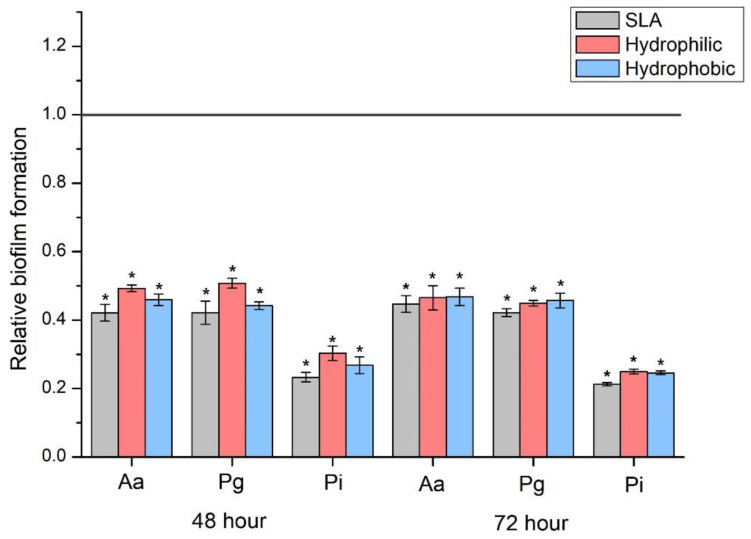
Comparison of relative biofilm formation ratios in relation to the machined group. * Significant differences between each group and machined group determined using the Mann-Whitney U-test, *p* < 0.05.

When the SLA, hydrophilic and hydrophobic groups were compared with the machined group as a reference, all three groups demonstrated a bacterial count ratio of less than 50% for all biofilm types (Figure 5) (*p* < 0.05).

## 4. Discussion

The present study investigated the influence of different surface treatments, specifically machined, SLA, hydrophilic (treated with a femtosecond laser), and hydrophobic (treated with a nanosecond laser), on dental Ti implant disk surface morphology, roughness, biofilm formation, and contact angle measurements. This study found that the hydrophobic group exhibited the highest surface roughness and a reduced bacterial count during the initial 48 h biofilm formation. The contact angle measurements also exhibited distinct differences between the hydrophilic and hydrophobic groups, suggesting that surface wettability played a role in bacterial adhesion and biofilm formation. However, at 72 h, the SLA, hydrophilic, and hydrophobic groups showed similar bacterial counts. Therefore, the hypothesis of this study was rejected (*p* < 0.05).

This study emphasizes that dental implant surface treatments can impact biofilm formation, ultimately affecting implant success. Previous research has demonstrated that increased surface roughness can enhance osseointegration while potentially promoting osteoblast formation [21,26]. On the other hand, in the present study, bacterial attachment was simulated by considering peri-implantitis following osseointegration. The present study supports these findings and further demonstrates that the inhibitory effect of the hydrophobic treatment using laser on initial biofilm growth aligns with those in existing literature [27], suggesting that surface properties can influence the bacterial adhesion and colonization. In the present study, the SLA and hydrophilic treatment groups showed no significant difference in surface roughness, with the Ra values having extremely low roughness below 2 µm (Table 3). Previous research has also demonstrated that the Ra values of the SLA surface were below 2 µm, which was reported to facilitate successful osseointegration [28]. Conversely, in the present study, the hydrophobic treatment group had significantly higher roughness, with Ra values exceeding 30 µm (Table 3). Future studies investigating the impact of such roughness on implant osseointegration are warranted and can potentially be advantageous as the transmucosal portion of the implant should provide a surface that promotes soft tissue attachment while inhibiting biofilm formation [8]. Additionally, this study employed confocal scanning microscopy, drawing on numerous prior studies [21,26,27,28], to assess surface roughness. However, given that atomic force microscopy can offer a more precise and comprehensive evaluation of surface roughness, further research utilizing atomic force microscopy is warranted.

Several previous studies have reported that various implant surface morphologies can cause significant differences in biofilm formation [4,6,7,9,11,12,13]. Furthermore, it has been emphasized that such a biofilm formation can have a significant impact on peri-implantitis [14]. In the present study, the hydrophilic treatment, involving the use of femtosecond laser technology, and the hydrophobic treatment, involving the use of nanosecond laser technology, have been demonstrated to exert a significant effect on bacterial adhesion. In addition, hydrophilic and hydrophobic treatments were performed using the laser treatment used in this study, and this was verified through the evaluation of the surface contact angle (Table 2, Figure 2). The divergent hydrophilic and hydrophobic properties induced by the different laser treatments can be attributed to the micro- and nano-scale topographical modifications that each laser type instigates on the titanium surface. These surface alterations directly influence the water contact angle, thus making the surface either hydrophilic or hydrophobic. Our study confirms these findings, underscoring the potential advantages of employing both femtosecond and nanosecond laser treatments for dental implant surface modification.

The results of this study suggest that surface treatment methods, particularly the hydrophobic treatment using nanosecond laser technology and the SLA treatment, can effectively modify dental implant surface treatments and reduce biofilm formation. The inhibitory effect of the hydrophobic treatment on initial biofilm growth (Pg and Pi) may be attributed to the unique surface properties created by the nanosecond laser, which may reduce bacterial adhesion and colonization. Furthermore, previous studies have indicated that the transmucosal part of the implant should provide a surface that inhibits biofilm formation while inducing soft tissue attachment [8,29]. These findings contribute to the understanding of how surface treatments can affect dental implant success and facilitate the development of advanced surface treatment technologies [30]. Although the hydrophobic treatment group in the present study exhibited significantly reduced initial biofilm growth than the SLA group, it did not yield superior results sufficient to replace the existing implant surface treatment, namely, SLA. These findings suggest that there is still room for further improvement and diversification of surface modification using nanosecond laser technology. The differences observed between the 48 and 24 h bacterial counts imply that the hydrophobic treatment may primarily affect the initial stages of biofilm formation. This is particularly relevant as early biofilm formation plays a crucial role in bacterial colonization and subsequent infection. Thus, the hydrophobic treatment may provide an additional advantage in reducing the risk of implant transmucosal part-associated infections.

This study demonstrates that hydrophobic treatment can inhibit the initial biofilm growth (Pg and Pi), thereby reducing bacterial adhesion and colonization. Prior research evaluated biofilm formation on titanium and zirconia implants through the counting of colonies of Aa, Pg, and Pi, and suggested potential for zirconia implants to mitigate peri-implantitis [31]. The biofilms of Aa, Pg, and Pi evaluated in this experiment significantly influence the incidence of peri-implantitis, with prior studies indicating a substantial correlation between the presence of Aa biofilm and peri-implantitis, independent of periodontal or mucosal health [32]. In the initial stages of biofilm growth, the Aa count was lower in the group treated with hydrophobic treatments than in the hydrophilic group, although biofilm growth was similar in both groups after 72 h. Ongoing studies are investigating methods to decrease biofilm formation and prevent or treat peri-implantitis, including techniques such as polymeric coatings of tobramycin, zinc, copper, silver, peptides, graphene oxide, titanium, and its alloys [33]. These methods have exhibited cytotoxicity against the biofilms of Aa, Pg, and Pi [34]. Further investigation is needed, not only into the impact of surface morphology on biofilm formation, but also into the potential application of diverse polymeric coatings as suggested in previous studies.

Surface wettability can be classified into hydrophobic (90° < θ < 150°), hydrophilic (10° < θ < 90°), superhydrophobic (θ > 150°), and superhydrophilic (θ < 10°) [18]. In previous studies, hierarchical structures were formed on Ti alloy surfaces using lasers, and various structures were used to confer hydrophilic and hydrophobic properties [16]. In the present study, the hydrophilic structures formed using femtosecond laser treatment demonstrated hydrophilic wettability (70.01°), whereas the hydrophobic structures formed using nanosecond laser treatment demonstrated hydrophobic wettability (112.12°). Wettability and surface energy are critical parameters for cell adhesion and spreading, with hydrophilic and hydrophobic surfaces being required for osseointegration and reduction of biofilm formation, respectively [30]. Consequently, it can be inferred that the surface wettability of hydrophobic structures formed using nanosecond laser treatment in the present study is consistent with the observed reduction in the initial biofilm growth compared with the other surface groups. Our study uncovered an intriguing correlation between surface wettability and bacterial growth: hydrophobic surfaces demonstrated a reduction in bacterial adhesion during the initial stages.

The present study has some limitations that need to be acknowledged. First, this study used an in vitro model, which may not fully replicate the complex in vivo environment of the oral cavity. Second, the sample size was relatively small, which could limit the generalizability of the findings. Third, this study focused on only three bacterial species, whereas dental biofilms are composed of a more diverse bacterial community. Finally, the long-term effects of the different surface treatments on biofilm formation and implant success were not evaluated. Future research should aim to overcome these limitations by using larger sample sizes, studying more diverse bacterial communities, and evaluating the long-term effects of surface treatments on biofilm formation and implant success. In vivo studies could provide a more comprehensive understanding of how different surface treatment methods influence dental implant performance in a clinical setting. Furthermore, exploring the combination of different surface treatments, such as hydrophilic and hydrophobic modifications, could reveal synergistic effects that could further increase the success rates of dental implants. Future studies will also need to include adhesion, proliferation, and differentiation of the osteoblast cells and cytocompatibility tests to ensure a comprehensive evaluation of the potential of these surface treatments.

## 5. Conclusions

In the present study, femtosecond and nanosecond lasers were successfully used to fabricate implant surface structures with hydrophilic and hydrophobic properties. The hydrophobic surface using nanosecond laser treatment had the highest surface roughness and influenced the reduced bacterial counts of Pg and Pi during the initial 48 h biofilm formation. In addition, the contact angle measurements showed differences in surface wettability between the groups, which could have contributed to the observed differences in biofilm formation. However, after 72 h of prolonged biofilm formation, the SLA, hydrophilic, and hydrophobic groups showed similar bacterial counts. This suggests that specific disk surface treatments formed through hydrophobic patterns using nanosecond laser treatment play an inhibitory role in the initial biofilm growth. Although the hydrophobic surface in the present study exhibited significantly reduced initial biofilm growth, it did not yield superior results sufficient to replace the existing implant surface treatment, namely, SLA. These findings suggest that there is still room for further improvement and diversification of surface modification using laser technology.

## Figures and Tables

**Table 1:** Comparison of the parameters for the hydrophilic and hydrophobic surface treatments.

Parameter	Hydrophilic Treatment	Hydrophobic Treatment
Laser type	Femtosecond laser	Nanosecond laser
Wavelength (nm)	343	355
Pulse duration	<400 fs	<30 ns
Power (W)	2~3	30
Repetition (kHz)	200	120
Speed (mm/s)	10	100
Lens	×10	Scanner

**Table 2:** Comparison of the surface contact angle on the different implant surface treatments.

Surface Treatment	Mean	SD	95% Confidence Interval		*p* *	Comparison **
			Lower	Upper		
Machined	75.04	1.80	73.75	76.33	<0.001	A
SLA	67.52	3.45	65.05	69.99		B
Hydrophilic	70.01	2.86	67.96	72.06		B
Hydrophobic	112.12	2.12	110.59	113.64		C

* Significant differences in the roughness of the different implant surface treatments determined using the Kruskal–Wallis H test, *p* < 0.05. ** Significant differences among the different implant surface treatments are indicated by different capital letters using the Bonferroni correction, *p* < 0.05.

**Table 3:** Comparison of the roughness (µm) on the different implant surface treatments.

Roughness Type	Surface Treatment	Mean	SD	95% Confidence Interval		*p* *	Comparison **
				Lower	Upper		
Ra	Machined	1.563	0.282	1.212	1.913	<0.001	A
	SLA	1.645	0.111	1.507	1.783		A
	Hydrophilic	0.903	0.241	0.603	1.203		A
	Hydrophobic	30.338	5.759	23.187	37.489		B
Rz	Machined	5.964	0.490	5.355	6.573	<0.001	A
	SLA	10.452	0.430	9.918	10.986		A
	Hydrophilic	7.335	1.539	5.424	9.246		A
	Hydrophobic	146.690	13.682	129.702	163.679		B

* Significant differences in the roughness of the different implant surface treatments determined using the Kruskal–Wallis H test, *p* < 0.05. ** Significant differences among the different implant surface treatments are indicated by different capital letters using Bonferroni correction, *p* < 0.05.

**Table 4:** Comparison of the viable bacterial counts (CFU/mL) of biofilms formed over 48 h on the four different implant surface treatments.

Biofilm Type	Surface Treatment	Mean	SD	95% Confidence Interval		*p* *	Comparison **
				Lower	Upper		
Aa	Machined	11,330	135	11,162	11,497	<0.001 *	A
	SLA	4770	228	4486	5053		B
	Hydrophilic	5580	83.6	5476	5683		C
	Hydrophobic	5200	136	5029	5370		D
Pg	Machined	9334	354	8894	9773	<0.001 *	A
	SLA	3928	193	3687	4168		B
	Hydrophilic	4738	111	4599	4876		C
	Hydrophobic	4122	94	4004	4239		B
Pi	Machined	19,390	1264	17,819	20,960	<0.001 *	A
	SLA	4500	45	4443	4556		B
	Hydrophilic	5860	253	5545	6174		C
	Hydrophobic	5178	169	4967	5388		B

* Significant differences in the viable bacterial counts on the different implant surface treatments determined using the Kruskal–Wallis H test, *p* < 0.05. ** Significant differences among the different implant surface treatments are indicated by different capital letters using Bonferroni correction, *p* < 0.05.

**Table 5:** Comparison of the viable bacterial counts (CFU/mL) of biofilms formed over 72 h on the four different implant surface treatments.

Biofilm Type	Surface Treatment	Mean	SD	95% Confidence Interval		*p* *	Comparison **	Comparison with 48 h *p* ***
				Lower	Upper			
Aa	Machined	11,984	173	11,768	12,199	<0.001 *	A	<0.001
	SLA	5353	256	5034	5672		B	0.005
	Hydrophilic	5573	394	5083	6063		B	0.972
	Hydrophobic	5605	270	5269	5941		B	0.017
Pg	Machined	9765	170	9554	9976	<0.001 *	A	0.041
	SLA	4119	73	4028	4210		B	0.092
	Hydrophilic	4390	53	4323	4457		B	0.001
	Hydrophobic	4461	178	4239	4682		B	0.006
Pi	Machined	23,703	977	22,488	24,917	<0.001 *	A	<0.001
	SLA	5039	209	4779	5299		B	0.004
	Hydrophilic	5904	113	5763	6045		B	0.734
	Hydrophobic	5817	157	5621	6013		B	<0.001

* Significant differences in the viable bacterial counts on the different implant surface treatments determined using the Kruskal–Wallis H test, *p* < 0.05. ** Significant differences among the different implant surface treatments are indicated by different capital letters using the Bonferroni correction, *p* < 0.05. *** Significant differences between 48 and 72 h using the Wilcoxon signed-rank test, *p* < 0.05.

## Data Availability

The datasets used and/or analyzed during the current study are available from the corresponding author on reasonable request.
